# The Great Imitator - Disseminated Tuberculosis Presenting as Baker’s Cyst: A Case Report

**DOI:** 10.5704/MOJ.2203.020

**Published:** 2022-03

**Authors:** A Shakya, N Patil, G Kakadiya, Y Soni

**Affiliations:** Department of Orthopaedics, Topiwala National Medical College, Mumbai, India

**Keywords:** tuberculous arthritis, pigmented villonodular synovitis, knee, delayed diagnosis, multifocal

## Abstract

Tuberculosis is known to be a great mimicker, and it can present in a myriad of ways, which often result in an incorrect diagnosis. In a country that is endemic to tuberculosis, the presentation can take many forms ranging from tumour to trauma. We present a case of Baker’s cyst that was provisionally diagnosed as pigmented villonodular synovitis (PVNS) of the knee and eventually turned out to be tuberculous arthritis. A 46-year-old male presented with an insidious swelling on the posterior aspect of his knee for one year. Magnetic resonance imaging was suggestive of PVNS as the likely diagnosis. The patient presented 21 days later with a foot drop. On following-up with further investigations, he was found to have a lesion at the level of the L4-L5 spine. Chest radiograph changes were suggestive of tuberculosis. A synovial biopsy of the knee was done, and the tuberculosis culture report was positive. The patient was started on anti-tubercular treatment and then operated on, with arthroscopic synovectomy and posterior open cyst excision. The histology report was positive for tuberculous synovitis. The patient completed the course of antitubercular drugs and had physiotherapy. He demonstrated a clinically and radiologically healed disease at the final follow-up with a good functional outcome. Clinicians must have a high index of suspicion for tuberculosis, especially in endemic areas. Getting a chest radiograph is recommended in every case. Early diagnosis with the appropriate treatment will give a good functional outcome for the patient.

## Introduction

There is a famous maxim in medicine that "uncommon presentations of common diseases are more common than common presentations of uncommon diseases," which is aptly applicable in the case of tuberculosis. Baker's cyst is a distention of the gastrocnemius-semimembranosus bursa commonly occurring secondary to a patellofemoralarthrosis^1,2^. Tuberculosis knee presenting as Baker's cyst is rare with only a few reports available and only one case of disseminated tuberculosis presenting as Baker’s cyst^1-4^.

We present a case that was provisionally diagnosed as PVNS but then turned out to be tuberculosis^4^.

## Case Report

A 46-year-old-male presented with an insidious onset of a swelling on the posterior aspect of his right knee for one year, which progressively increased to reach a dimension of 14cm x 6cm x 5cm. There was no history of trauma, fever, or comorbidities. However, he had pain and restriction of movement and could not carry out his daily activities and bear weight on the affected limb. On general examination, the patient was malnourished and afebrile. Clinically, the swelling was warm, fluctuant, compressible with a positive patellar tap test but without transillumination, pulsatility, tenderness, erythema, and reducibility.

**Fig. 1:**
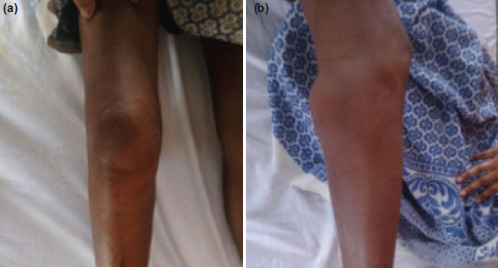
Clinical photo of the right knee. (a) Anterior view of the knee with slight swelling over the medial aspect of the knee. (b) Right lateral view of the knee with an obvious fullness over the posterior aspect of the knee predominantly below the knee crease with minimal swelling anteriorly.

**Fig. 2:**
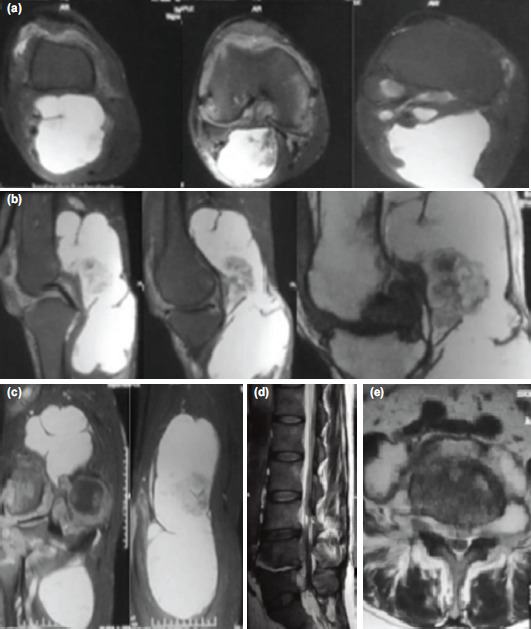
MR images of the knee and lumbosacral spine. (a) Axial cut T2 PDS view of the right knee showing a hyperintense mass located posteriorly popliteal fossa (b) Sagittal cut T2 and T1 images of the right knee showing the same hyperintense mass extending completely in the popliteal fossa with a hypointense lesion in the middle suggestive of clumps of hemosiderin deposition (red arrow). (c) Coronal cut T2 images of the right knee indicative of the same lesion and its continuous extent in the popliteal fossa with a probable frond of synovial tissue in the cyst. (d) Sagittal cut T2 images of the lumbosacral spine showing a typical tuberculous paradiscal lesion at L4-L5 level. (e) Axial cut images of the lumbar spine at L4-L5 level suggestive of cord compression due to tuberculous lesion and associated bony destruction.

The range of motion was from 10° extension to 90° flexion, which was painful with a soft endpoint (Fig. 1). A provisional diagnosis of Baker's cyst was made. The radiograph was normal apart from the soft tissue shadow posteriorly. MRI was done, which was suggestive of PVNS (Fig. 2).

Ultrasonography of the knee joint showed a hypoechoic-heterogenous collection with synovial hypertrophy. A total of 225cc of fluid was found in the cyst with no joint communication, and was aspirated and analysed (Table I). The leucocyte count was 7510/μL (Neutrophils=78.8%, Lymphocytes=16.97%), and the renal and liver function tests were normal. Erythrocyte sedimentation rate (ESR) was 110mm at the end of one hour, and C-Reactive Protein (CRP) was 12mg/dl (>1mg/dl = significant inflammation). The test for the human immunodeficiency virus (HIV) was negative.

**Table I:** Results of the synovial biopsy and fluid analysis

Sample	S. No.	Test	Result
Synovial tissue	1.	Gram Stain	Negative
	2.	Culture of aerobes and anaerobes	Negative
	3.	Mycobacterium tuberculosis staining and Culture	Positive
	4.	Histopathology	Granulomas
Synovial fluid	1.	Gram stain	Negative
	2.	Culture of aerobes and anaerobes	Negative
	3.	Mycobacterium tuberculosis staining and Culture	Positive
	4.	WBCs	45000/mm3
	5.	GenXpert	Positive with no rifampicin resistance
	6.	ADA level	Negative (<34U/L)

Three weeks later, he developed an acute onset of right extensor hallucis longus (EHL) and ankle weakness with a power of the Medical Research Council (MRC) grade 3. A lumbosacral spine radiograph showed an L4-L5 paradiscal lesion. Chest radiograph was suggestive of miliary tuberculosis. He was isolated, and all necessary precautions were taken. A magnetic resonance imaging (MRI) of the lumbosacral spine suggested tuberculosis at the L4-L5 spinal level (Fig. 2). Sputum acid-fast bacilli (AFB) staining turned out to be positive. He was started on empirical antituberculous treatment (ATT) (Isoniazid 225mg, Rifampicin 450mg, Pyrazinamide 900mg, and Ethambutol 675mg). Tuberculosis evaluations of the family members and close contacts were negative. A computed tomography (CT) guided biopsy of the L4-L5 level was done, and the sample was sent for analysis. The six-week tuberculosis culture report of both the synovial and spine biopsies turned out positive with sensitivity to all the first-line medications. After a month of ATT, sputum AFB was negative thrice. The patient's general condition improved, and he was posted for anterior arthroscopic synovectomy and posterior open cyst excision with appropriate precautions.

**Fig. 3:**
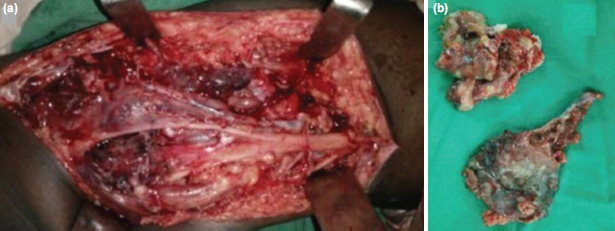
(a) The posterior approach to knee showing the carefully dissected neurovascular bundle with interposed tuberculous cyst. (b) The excised reddish brown tuberculous cyst.

Under general anaesthesia, an arthroscopic synovectomy was performed in the supine position. The patient was then turned prone, and the posterior approach was taken with a lazy S-shaped incision (proximal lateral and distal medial). The sural nerve and the common peroneal nerve were identified and retracted (Fig. 3). The tissue was dirty reddish-brown. The cyst was thoroughly excised, and its communication with the posterior capsule cut and repaired using a purse-string suture. The wound was then closed in layers with a drain in situ.

Post-operatively, the lower limb was splinted in 20° of knee flexion to avoid tension on the suture line for five days, after which the range of motion of knee exercises was started. Histology report showed Langhan's giant cells and epithelioid granuloma. The intensive phase of ATT was continued for three months, during which time the EHL and ankle power improved to MRC grade 5. At the final follow-up, the patient had completed 18 months of ATT and was pain-free with a good range of movement.

## Discussion

Skeletal tuberculosis accounts for less than 2% of all tuberculosis cases, with the spine, hip, and knee the most common sites for tuberculosis^1,5^. Multifocal skeletal tuberculosis is uncommon, and the diagnosis is frequently delayed^4^.

A myriad of tests for diagnosis of tuberculosis has emerged like AFB staining, GeneXpert, culture on Lowenstein-Jensen medium, Mycobacteria Growth Indicator Tube (MGIT), and histopathology. Skeletal tuberculosis is paucibacillary, and none of the rapid-detecting tests have high sensitivity or specificity. Culture is the gold standard but requires a long period (up to eight weeks). Thus, the onus is on histopathology to detect granuloma and caseous necrosis with a yield of 90%^3^.

Knee tuberculosis classically presents as knee arthritis or synovitis, of which Baker's Cyst is a rare presentation^2^. On MRI, PVNS, as well as tuberculosis, may show similar findings. A delay in diagnosing either PVNS or TB can result in widespread joint destruction. Establishing the diagnosis is important as the treatment algorithm for either condition is completely different^5^. Since we maintained a high clinical index of suspicion, we could get a positive result for tuberculosis culture.

Thus, establishing a proper protocol for investigation is a must. At our institute, we routinely investigate all cases of swelling with a Gram stain, aerobic and anaerobic cultures, AFB staining and culture, histopathology, and GeneXpert. In a case of a fluctuant swelling, the fluid is tested for a Gram stain, aerobic and anaerobic cultures, AFB staining and culture, leucocyte count, GeneXpert, and synovial fluid adenosine deaminase (ADA) levels.

In conclusion, clinicians must have a high index of suspicion for tuberculosis, especially in the endemic areas. A chest radiograph is recommended in every case of a swelling as it can render the rare causes and expensive management unnecessary. A sound clinical and diagnostic approach is necessary for getting the right diagnosis.
